# Quinazoline-based *α*1-adrenoceptor antagonists induce prostate cancer cell apoptosis via TGF-*β* signalling and I*κ*B*α* induction

**DOI:** 10.1038/sj.bjc.6600961

**Published:** 2003-05-13

**Authors:** J V Partin, I E Anglin, N Kyprianou

**Affiliations:** 1 Division of Urology, Department of Surgery, University of Kentucky Medical Center, Lexington, KY 40536, USA; 2 Department of Molecular Biochemistry, University of Kentucky Medical Center, Lexington, KY 40536, USA

**Keywords:** prostate cancer, apoptosis, quinazolines, *α*1-adrenoceptor antagonists, Smad, TGF-*β*1, caspase-3, I*κ*B*α*

## Abstract

Previous studies documented the ability of quinazoline-based *α*1-adrenoceptor antagonists to induce apoptosis in prostate cancer cells via an *α*1-adrenoceptor-independent mechanism. In this study we investigated the molecular events initiating this apoptotic effect. Since transforming growth factor-*β*1 (TGF-*β*1) mediates prostate epithelial cell apoptosis, we hypothesised that the activation of the TGF-*β*1 pathway underlies the quinazoline-based apoptotic effect in prostate cancer cells. Treatment of the androgen-independent human prostate cancer cells PC-3 with doxazosin resulted in a strong caspase-3 activation within 24 h, whereas tamsulosin, a sulphonamide-based *α*1-adrenoceptor antagonist, had no significant apoptotic effect against prostate cancer cells. To identify the molecular components involved in this quinazoline-mediated apoptosis, cDNA microarray analysis of PC-3 prostate cancer cells treated with doxazosin (3 h) was performed. Induced expression of several genes was observed including p21^WAF-1^ and I*κ*B*α* (inhibitor of NF-*κ*B alpha). Relative quantitative reverse transcription–polymerase chain reaction analysis revealed induction of several TGF-*β*1 signalling effectors: Induction of mRNA for Smad4 and the TGF-*β*1-regulated apoptosis-inducing transcription factor TGF-*β*1-inducible early gene (TIEG1) was detected within the first 6 h of doxazosin treatment. Upregulation of I*κ*B*α* at both the mRNA and protein level was also detected after 6 h of treatment. Furthermore, doxazosin resulted in a considerable elevation in Smad4 and TIEG protein expression (6 h). A ‘latent’ increase in TGF-*β* mRNA expression was detected after 48 h of treatment. These findings suggest that the quinazoline-based doxazosin mediates prostate cancer apoptosis by initially inducing the expression of TGF-*β*1 signalling effectors and subsequently I*κ*B*α*. The present study provides an initial insight into the molecular targets of the apoptotic action of quinazolines against prostate cancer cells.

Prostate cancer continues to afflict many thousands of American males and is the cause of approximately 30 000 deaths per year (Greenlee *et al*, 2000). Although therapeutic modalities such as androgen ablation and radical prostatectomy are considered curative for localised disease, no treatment for metastatic prostate cancer is available, which shows a significant increase in length of life (Raghavan, 1988; Crawford *et al*, 1989; Catalona *et al*, 1993). The therapeutic significance of apoptosis in the treatment of prostate cancer emerges from evidence suggesting that like normal prostate epithelial cells, prostate cancer cells maintain sensitivity to androgens and undergo apoptosis in response to androgen withdrawal (Isaacs, 1994; Boyle, 2000). Androgen-independent prostate cancer cells still contain the apoptotic machinery and can undergo apoptosis in response to hormone-independent approaches (Bruckheimer and Kyprianou, 2000). Reactivation of cell death pathways in prostate cancer cells represents a powerful approach for pharmacological intervention, and consequently targeting the apoptotic signalling in prostate cells has been the focus of numerous investigations (Bruckheimer and Kyprianou, 2000).

A class of pharmacological agents, the *α*1-adrenoceptor antagonists, originally used as antihypertensive agents (Young and Brogden, 1988), have been safely used as standard medical therapy for benign prostatic hyperplasia (BPH) and the long-term relief of lower urinary tract symptoms (LUTS) (Caine, 1988; Kirby, 1996). Growing evidence suggests that two of these clinically used *α*1-adrenoceptor antagonists, doxazosin and terazosin, that share a similar chemical structure, the quinazoline nucleus (Figure 1

**Figure 1:**
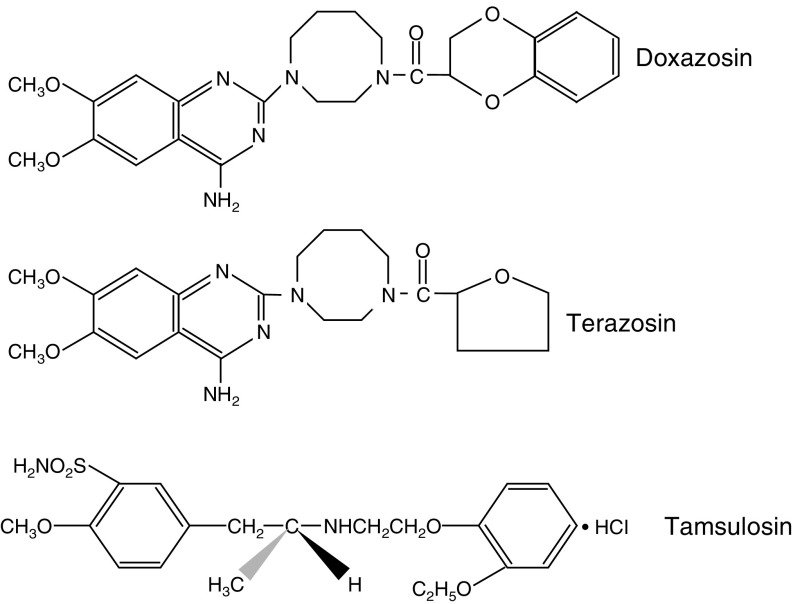
Chemical structure of *α*1-adrenoceptor anatagonists. The quinazoline nucleus is shared by the two quinazoline-based *α*1-adrenoceptor antagonists doxazosin and terazosin, but not by the methoxysulphonamide-derived *α*1-adrenoceptor antagonist tamsulosin.

), exert a potent apoptotic effect against prostate cancer cells (Kyprianou and Benning, 2000), via an *α*1-adrenoceptor-independent mechanism (Benning and Kyprianou, 2002) and suppression of tumour vascularity (Keledjian *et al*, 2001).

Apoptosis induction by androgen withdrawal in normal and hormone-dependent malignant prostate involves the transforming growth factor-*β*1 (TGF-*β*1) signalling pathway (Kyprianou and Isaacs, 1989). Recent immunohistochemical analysis of BPH tissue revealed an increase in TGF-*β*1 expression but no change in TGF-*β*II receptor (T*β*RII) in patients who had undergone treatment with the clinically used quinazoline-based *α*1-adrenoceptor antagonist terazosin (Glassman *et al*, 2001). The TGF-*β*1 signalling system normally functions to inhibit proliferation and induce apoptosis of epithelial and endothelial cells (Massague *et al*, 2000); its signal transduction pathway involves the receptor-mediated activation by phosphorylation of the intracellular effectors, Smad2, Smad3 and Smad4, and their translocation to the nucleus where they regulate gene transcription (Massague, 2000). Expression and activation of these intracellular effectors of the TGF-*β*1 pathway are critical to the execution of the apoptotic process (DeCaestecker *et al*, 2000). An inhibitory regulator of TGF-*β*1 signalling, Smad7, (Whitman, 1997) is regulated by TGF-*β*1 specifically via Smad3 and Smad4 indicating a negative feedback mechanism (Hayashi *et al*, 1997). Smad7 is induced during apoptosis of prostate epithelial cells (Brodin *et al*, 1999) and has recently been shown to sensitise other cell types to apoptosis (Mazars *et al*, 2001; Schiffer *et al*, 2001).

The TGF-*β*1-inducible early gene (TIEG1) is another factor that induces apoptosis in breast, lung and kidney cells (Tachibana *et al*, 1997; Chalaux *et al*, 1999; Hefferan *et al*, 2000), potentially by downregulating the antiapoptotic protein bcl-2 (Chalaux *et al*, 1999).

This study aimed to identify the molecular mechanism underlying the apoptotic action of quinazoline-based *α*1-adrenoceptor antagonists against prostate cancer cells. Our findings demonstrate that doxazosin induces the apoptotic signalling potentially via the activation of TGF-*β*1 pathway with a potential involvement of NF-*κ*B nuclear effectors.

## MATERIALS AND METHODS

### Materials

The *α*1-adrenoceptor antagonists used in this study were generously provided by the following pharmaceutical companies: doxazosin (Cardura; Doxazosin Mesylate) was provided by Pfizer Pharmaceuticals (New York, NY, USA); terazosin (Hytrin; terazosin hydrochloride) was obtained from Abbott Laboratories (Abbot Park, IL, USA); and tamsulosin (Flomax) was provided by Yamanouchi Pharmaceuticals (Tokyo, Japan).

### Cell culture

The human prostate cancer PC-3 cell line was obtained from the American Type Tissue Culture Collection (Rockville, MD, USA). PC-3 cells were routinely maintained in RPMI (Life Technologies, Gaithersburg, MD, USA), containing fetal bovine serum (FBS) (10%) and antibiotics.

### cDNA microarray analysis

Microarray analyses were performed according to the manufacturer's instructions. Microarray filters (SuperArray Inc., Bethesda, MD, USA) consisting of 23 markers for specific signal transduction pathways including mitogenic (Egr-1, c-*fos*), antiproliferative (p19, p21^Waf1^) and p53 (mdm2, bax) pathways were used. Briefly, total RNA was isolated from both untreated PC-3 cells (control) or cells treated with doxazosin (25 *μ*M) for 3 h. Biotin-labelled probes were made from the isolated control and test RNA. The filters were exposed to film and the genes were visualised as dots. A score for the relative intensities of these genes was calculated after densitometric analysis using the Scion Image program (National Institutes of Health, USA, http://rsb.info.nih.gov/scion-image/). Equal probe hybridisation to each filter was confirmed by *β*-actin and GAPDH internal controls. Results represent the mean of two independent experiments.

### RT–PCR analysis

Exponentially growing cultures of PC-3 cells were treated with doxazosin (25 *μ*M) for 6, 12, 24 and 48 h. Total cellular RNA was isolated from untreated control and treated cells using the Trizol reagent. RNA (1 *μ*g) was reverse transcribed into cDNA using the SuperScript II system (GIBCO BRL, Grand Island, NY, USA) in a thermocycler (Biometra, Göttingen, Germany). The RNA, dNTPs and random hexamers were incubated for 5 min at 65°C followed by the addition of the buffer, reverse transcriptase (RT), Rnase inhibitor and MgCl_2_, and the reaction mixture was incubated at 42°C for 50 min. The polymerase chain reaction for TGF*β*, T*β*RII, Smad4, Smad7 and TIEG was performed in a total volume of 25 *μ*l containing 0.5 *μ*l RT reaction mixture, 50 mM KCl, 10 mM Tris-HCl, 2 mM MgCl_2_, 2.5 U *Taq* DNA polymerase, 0.15 *μ*M of each forward and reverse primer, and distilled water. The following cycling conditions were used for 37 cycles in a thermal cycler (Perkin-Elmer Gene Amp 2400 Wellesley, MA, USA or Biometra T Gradient, Gottingen, Germany): initial denaturation, 5 min at 94°C; denaturation 30 s at 94°C; annealing 30 s at 55°C and elongation, 30 s at 72°C. The programme was followed by a final elongation step for 5 min at 72°C. Control reactions were performed for each series by omitting either the mRNA or RT. For qualitative analysis, RT–PCR products were electrophoresed through agarose gels (2.5%) and visualised with ethidium bromide staining.

Relative quantitative RT–PCR was performed with the QuantumRNA™ 18S Internal Standards kit (Ambion Austin, TX, USA) according to the protocol recommended by the manufacturer with some modifications. Briefly, the ratio of 18S rRNA primers to competimers that resulted in a PCR product approximately equal to abundance of the specific gene at the selected number of cycles of amplification was determined. The recommended 18S primer: competimer ratios of 1 : 9, 2 : 8 and 3 : 7 were tested and a 1 : 9 ratio resulted in an approximately equal ratio for all four products. Relative quantitative RT–PCR was subsequently performed using RNA from untreated control and doxazosin-treated cells. Samples (10 *μ*l) of PCR products were subjected to electrophoretic analysis on agarose gels (1.8% w v^−1^) and bands were visualised with SYBR Gold nucleic acid stain (Molecular Probes, Eugene, OR, USA). Densitometric analysis was performed using a UVP Bioimaging System (Upland, CA, USA). The expression profile of the various genes after doxazosin treatment was analysed in three independent experiments.

### Western blot analysis

Cells from untreated control and treated cells (with either doxazosin or tamsulosin) were lysed in RIPA buffer (150 mM NaCl, 50 mM Tris pH 8.0, 0.5% deoxycholic acid: 1% Nonidet P40 with 1 mM PMSF). Total cell lysates (30–50 *μ*g) were subjected to sodium dodecyl sulphate (SDS)–polyacrylamide gel electrophoresis, then transferred onto Hybond-P membranes (Amersham Pharmacia Biotech., Piscataway, NJ, USA). Membranes were blocked with 5% dry milk in TBS-T (Tris-buffered-saline containing 0.05% Tween-20) for 1 h at room temperature, and were subsequently incubated with the primary antibody (18 h at 4°C). Expression of specific proteins was assessed using the respective antibodies: Smad4 (Oncogene, Boston, MA, USA), caspase-3 (Pharmigen, San Diego, CA, USA), I*κ*B*α* (Santa Cruz Biotechnology, Santa Cruz, CA, USA), TIEG1 (a generous gift from Dr R Urrutia, Mayo Clinic, Rochester, MN, USA) and *α*-actin was from Calbiochem #CP01; San Diego, CA, USA). Following incubation with the respective primary antibody, membranes were incubated with species-specific horseradish peroxidase (HRP)-labelled secondary antibodies (1 h at room temperature). Membranes were subsequently incubated with the enhanced chemiluminescence system (ECL, Amersham Life Sciences RPN 2108) and autoradiographed using X-ray film (Amersham Pharmacia Biotech). Densitometric analysis was performed using the Scion Image Program (National Institutes of Health, USA, http://rsb.info.nih.gov/scion-image). Bands were normalised to *α*-actin expression and shown as fold changes relative to untreated controls.

### ELISA

The procedure was essentially performed according to the manufacturer's instructions (Promega, WI, USA). Briefly, a 96-well plate was coated with TGF-*β*1 antibody in carbonate coating buffer and incubated overnight at 4°C. Supernatants derived from PC-3 cells after various periods of doxazosin treatment were added to the respective wells (0, 3, 6, 9 and 24 h). Secondary anti-TGF-*β*1 (1 : 1000 dilution) was added to the samples and incubated for 2 h. Plates were subsequently washed and TGF-*β*1–horseradish peroxidase (HRP) conjugate was added. Upon termination of the reaction, the absorbance was read at *A*_450_.

## RESULTS

**Figure 2:**
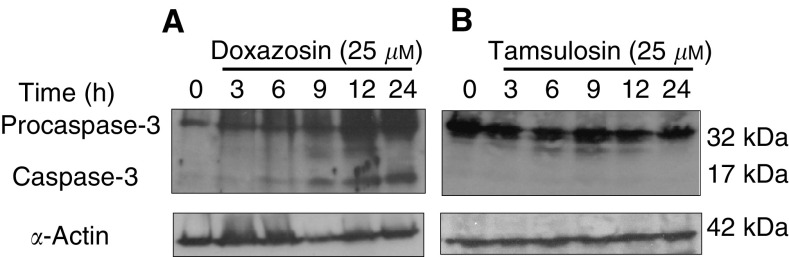
Effect of doxazosin on caspase-3 activation in prostate cancer cells. PC-3 prostate cancer cells were cultured and treated with either doxazosin (25 *μ*M) or tamsulosin (25 *μ*M) for 0, 3, 6, 9, 12 and 24 h. Cell lysates (40 *μ*g protein) were subjected to Western blotting as described in ‘Materials and Methods’. The expression and activation of caspase-3 in response to doxazosin (panel **A**) and tamsulosin (panel **B**) treatment is shown. Molecular weight sizes are indicated on the right.

Previous studies have demonstrated that the piperizidinyl quinazoline-based *α*1-adrenoceptor antagonists doxazosin and terazosin (Figure 1) induce apoptosis in prostate cancer cells, whereas the methoxysulphonamide-derived *α*1-adrenoceptor antagonist tamsulosin had no apoptotic function (Kyprianou and Benning, 2000). Caspases as apoptosis executioners upon sequential activation can cleave protein substrates leading to apoptotic cell death (Cohen, 1997). To determine the involvement of the caspase cascade in *α*1-adrenoceptor antagonist-mediated apoptosis and the observed differences in apoptotic capability between these two structurally dissimilar drugs, we investigated the activation of caspase-3 in prostate cancer cells treated with either doxazosin or tamsulosin. Western blotting revealed the temporal induction of caspase-3 (17 kDa) in doxazosin-treated cells after 9 h, an effect that was maintained up to 24 h (Figure 2A

). The activated form of caspase-3 was not detected in tamsulosin-treated prostate cancer cells (Figure 2B), consistent with the inability of this compound to induce apoptosis (Kyprianou and Benning, 2000).

In order to determine the early alterations in gene expression that may be causally involved in quinazoline-induced apoptosis, cDNA microarray analyses were performed. RNA was isolated from cultures of PC-3 cells, untreated control or following treatment with doxazosin (25 *μ*M) for 3 h. Table 1

**Table 1:** Microarray analysis of doxazosin-mediated gene expression in prostate cancer cells

**Gene**	**Genebank ID**	**Relative gene expression**
ATF-2 (creb-2)	X15875	0.1
Bax	L22474	0.6
CD5	X04391	1.3
c-*fos*	V01512	0.5
c-*myc*	J00120	1.8
Cyp19 (aromatase p450)	M28420	0.1
Egr-1	X52541	0.4
**Fas ligand**	**U08137**	**3.3**
Gadd45	M60974	1.3
**Hsf-1 (tcf5)**	**M64673**	**4.4**
Hsp27 (hsp b1)	Z23090	1.6
Hsp90 (CDw52)	X15183	1.7
**i*κ*B*α* (mad3)**	**M69043**	**8.9**
IL-2	U25676	1.9
iNOS	L09210	0.6
Mdm2	Z12020	2.1
NF*κ* B	M58603	3.2
p19	U40343	2.5
**p21^WAF-1^**	L47233	**7.2**
p53	M14694	NA
p57 (Kip2)	U22398	1.2

NA=not applicable. PC-3 cells were treated with doxazosin (25 *μ*M) for 3 h and subjected to microarray analysis as described in ‘Experimental Procedures’. Data represent the mean scores of duplicate genes on each membrane from two independent experiments. Values are expressed as the relative number of doxazosin-treated *vs* untreated (control) cells.

summarises the expression profile of doxazosin-regulated genes in human prostate cancer cells. Out of the 23 genes examined, eight were upregulated by at least two-fold, whereas four were downregulated. Our array analysis revealed a dramatic induction of two genes within 3 h of doxazosin treatment p21 (seven-fold) and I*κ*B*α* (nine-fold). Interestingly, both these genes are inducible by TGF-*β*1 (DeCaestecker *et al*, 2000; Massague, 2000), suggesting a potential reactivation of this signalling pathway by doxazosin. Two other apoptosis-regulating genes were also moderately induced, the fas ligand (>3-fold) and hsf1 (four-fold).

**Figure 3:**
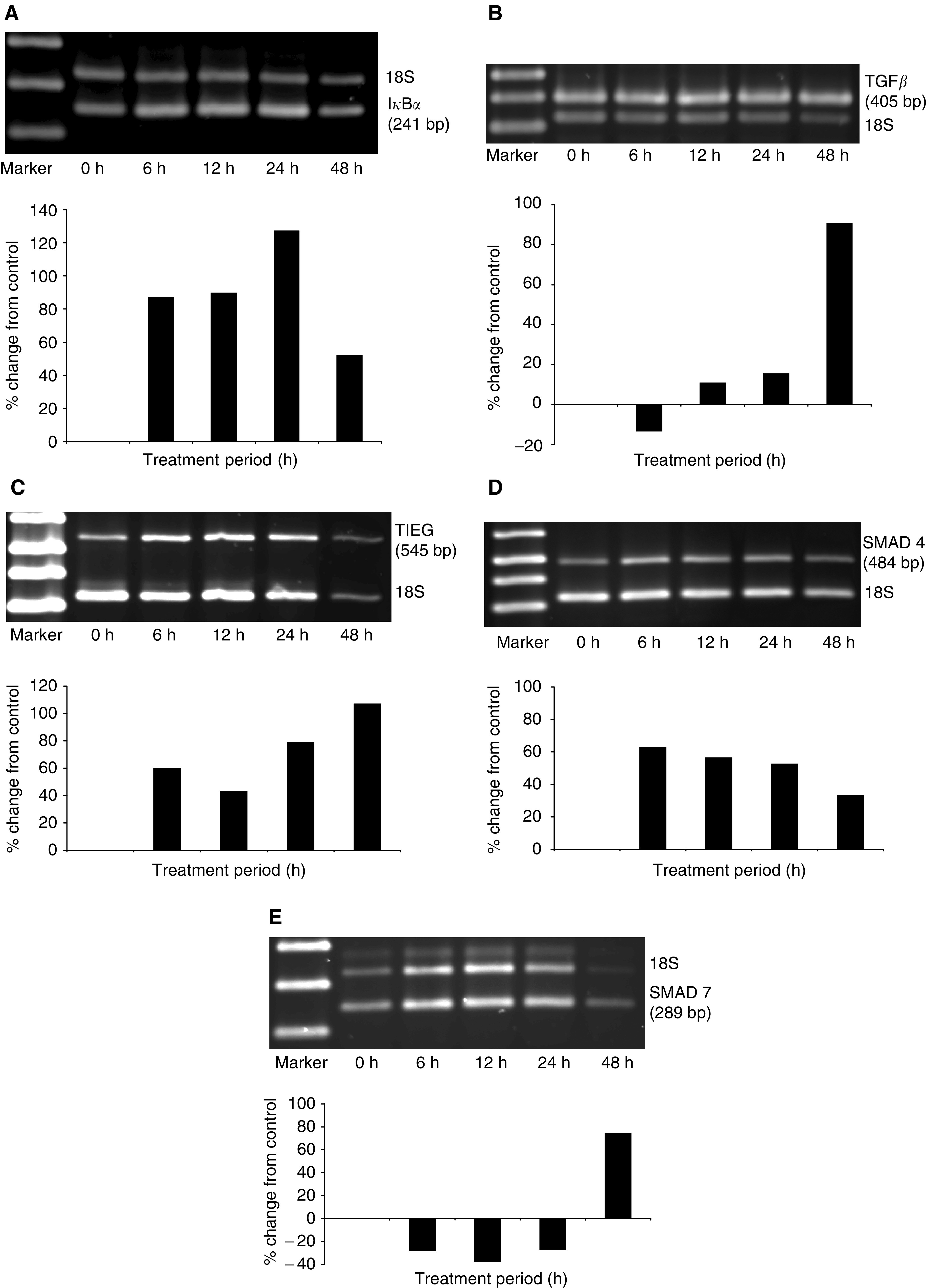
Quantitative RT–PCR analysis of mRNA expression for TGF-*β*1 signalling pathway effectors in response to doxazosin. PC-3 prostate cancer cells were treated with doxazosin (25 *μ*M) for 6, 12, 24 and 48 h. Total RNA was isolated from untreated control and doxazosin-treated cells and subjected to RT–PCR with primers specific for I*κ*B*α* (panel **A**), TGF*β* (panel **B**), TIEG 1 (panel **C**), Smad4 (panel **D**), Smad7 (panel **E**), and relative quantitative RT–PCR was performed as described in ‘Materials and Methods’. This figure is the representative of three independent experiments. The molecular weights for the specific gene and the 18S products are shown on the right. The marker was a 1 kb plus DNA ladder (GIBCO BRL).

**Figure 4:**
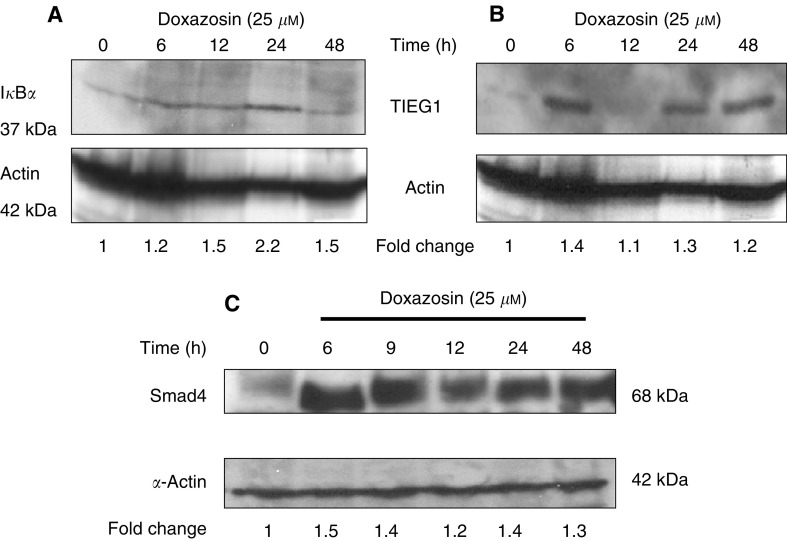
Effect of doxazosin on I*κ*B*α*, TIEG1 and Smad4 protein expression in prostate cancer cells. PC-3 prostate cancer cells were cultured and treated with doxazosin (25 *μ*M) for times as indicated (0–24 h). Cell lysates were isolated from treated and untreated cells and subjected to Western blot analysis. Protein expression of I*κ*B*α* (**A**), TIEG1 (**B**) and Smad4 (**C**) were examined by Western blotting using the respective antibodies as described in ‘Materials and Methods’. Expression of *α*-actin was used as a normalising control and fold changes for each specific protein relative to untreated controls (after normalisation to *α*-actin expression) are shown.

Since induced expression of I*κ*B*α* was detected within 3 h of doxazosin treatment (Table 1), we examined the mRNA and protein expression profile in PC-3 cells over a 48-h treatment period with the drug. The RT–PCR analysis revealed a significant doxazosin-mediated induction in mRNA expression within 6 h (approx. 100% relative to untreated control cells); a maximum increase in I*κ*B*α* mRNA levels was detected after 24 h and at 48 h of treatment a moderate decrease was observed (Figure 3A

). A parallel temporal elevation of I*κ*B*α* protein expression reaching a maximum after 24 h of doxazosin treatment was detected by Western blot analysis (Figure 4A

).

**Figure 5:**
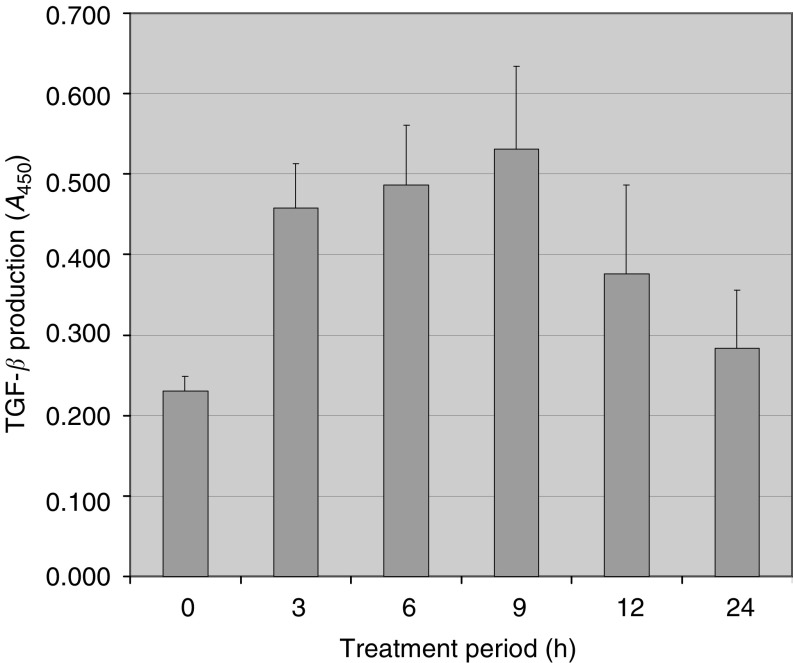
Effect of doxazosin on TGF-*β*1 protein secretion by prostate cancer cells. PC-3 prostate cancer cells were treated with doxazosin (25 *μ*M) for various periods of time as indicated (0–24 h). Supernatants were obtained from treated cells and subjected to ELISA as described under ‘Materials and Methods’. Values represent the mean of three independent experiments performed in duplicate±standard deviation (s.d.).

To gain a further insight into the mechanism underlying quinazoline-induced apoptosis, the temporal expression of several genes involved in TGF-*β*1-mediated apoptosis, that is, the ligand TGF-*β*, its critical receptor T*β*RII receptor, the intracellular effectors Smad4 and Smad7, and the nuclear factor TIEG1 was evaluated. Since increased TGF-*β*1 protein expression is associated with apoptosis (Glassman *et al*, 2001), we first examined the expression profile of TGF-*β*1 in doxazosin-treated prostate cancer cells. A significant increase in TGF-*β*1 mRNA expression were detected within the first 12 h of doxazosin treatment (Figure 3B). This mRNA induction for TGF-*β* resulted in a significant increase in the levels of active TGF-*β*1 protein produced by PC-3 prostate cancer cells (*P*<0.05). After 24 h of exposure to doxazosin, there was a decline in TGF-*β*1 levels to baseline values (Figure 5

). No changes in the T*β*RII receptor mRNA expression by PC-3 cells were observed in response to doxazosin (data not shown).

A marked induction in TIEG1 (Figure 3C) and Smad4 (Figure 3D) mRNA levels was observed within 6 h of treatment (65% increase), while there was a transient decrease in Smad7 mRNA expression for 24 h of doxazosin exposure, and it was subsequently followed by an upregulation by 48 h compared to the untreated control PC-3 cells (Figure 3E). Smad4 is an important regulator of TGF-*β*1 signalling and apoptosis in a variety of cell lines (Kim *et al*, 1996; Liu *et al*, 1997). Considering that Smad4 mRNA is upregulated, we investigated the effect of doxazosin on Smad4 protein levels. As shown in Figure 4C, there was an increase in Smad4 protein expression in response to doxazosin and this elevation was maintained throughout all treatment periods examined (up to 24 h), a pattern that temporally correlates with the mRNA expression profile (Figure 3D).

## DISCUSSION

The ability of quinazoline-derived *α*1-adrenoceptor antagonists to induce apoptosis in human prostate cancer cells via an *α*1-adrenoceptor-independent mechanism has been established (Kyprianou and Benning, 2000; Benning and Kyprianou, 2002). The present study focused on dissecting the molecular mechanism underlying this action. Our findings demonstrate that caspase-3 was activated in response to doxazosin and not tamsulosin, evidence confirming the apoptotic nature of the action of quinazoline-derived *α*1-adrenoceptor antagonists against prostate cell growth. This induction was first evident within 9 h of treatment and was maintained up to 24 h, a pattern that temporally correlates with the morphological manifestations of apoptosis in prostate cancer cells following doxazosin treatment (Kyprianou and Benning, 2000).

TGF-*β*1 has recently been shown to activate caspase-3 to induce apoptosis in the NRP-154 prostate epithelial cell line (Chipuk *et al*, 2001), evidence supporting our hypothesis that doxazosin may activate apoptosis of prostate cancer cells through TGF-*β*1 signalling. Doxazosin resulted in a ‘latent’ induction of TGF-*β*1 mRNA expression that was subsequent to a moderate increase in the active TGF-*β*1 levels by prostate cancer cells PC-3 (after 24 h of treatment). While a similar pattern has been exhibited by apoptotic T-cells with an enhanced release of active TGF-*β*1 without major changes in the mRNA expression (Chen *et al*, 2001), one may also consider the possibility that the observed upregulation of TGF-*β* mRNA in prostate cancer cells could be a consequence of doxazosin-induced apoptosis. The lack of induction of T*β*RII expression in prostate cancer cells by doxazosin is consistent with our earlier report indicating no changes in T*β*RII protein levels in prostate tissue (from BPH patients) after treatment with the quinazolines (Glassman *et al*, 2001). It appears that doxazosin modulated the expression of the intracellular effectors TGF-*β*1 signalling pathway, Smad4 and Smad7 proteins, towards activation of the apoptotic machinery.

Microarray-based examination of doxazosin mediated-gene expression revealed the rapid upregulation of two TGF-*β*1-modulated genes, I*κ*B*α* and p21^WAF-1^. Doxazosin-induced over-expression of I*κ*B*α* may have implications for NF-*κ*B intracellular signalling. For instance, in prostate cancer cells and metastatic melanoma cells, the blockade of NF-*κ*B by a nondegradable dominant-negative I*κ*B*α* mutant suppresses angiogenesis, invasion and metastasis (Huang *et al*, 2000,^2001^), and may reduce the apoptotic effects associated with NF-*κ*B signalling (Baldwin, 1996). These reports together with the observed upregulation of I*κ*B*α* in prostate cancer cells may provide a mechanism to explain the increased apoptosis and reduced tumour vascularity in prostate cancer specimens (Keledjian *et al*, 2001). Constitutive activation of NF-*κ*B has been reported in many tumours (Baldwin, 1996), including androgen-independent prostate cancer cells (Pajonk *et al*, 1999; Palayoor *et al*, 1999; Suh *et al*, 2002) and oestrogen receptor-negative breast cancer cells (Biswas *et al*, 2001). This activation of NF-*κ*B survival signalling is believed to provide a growth advantage in androgen-independent prostate cancer cells by suppressing apoptosis and inducing cell adhesion and angiogenic potential (Huang *et al*, 2001; Gasparian *et al*, 2002).

Smad4 is the principal intracellular effector of TGF-*β*1 signalling and its expression is regulated at the protein and mRNA level by doxazosin in PC-3 cells. The mechanism may involve the inhibition of Smad4 degradation, recently reported to be mediated by the oncogenic Ras pathway. Blockade of the oncogenic Ras pathway using a quinazoline-based tyrosine kinase inhibitor (TKI) PD98059 restores Smad4 expression levels and TGF-*β*1 signalling *in vitro* (Saha *et al*, 2001). Upregulation of Smad4 would also facilitate the transcription of several TGF-*β*1-regulated genes such as Smad7 (Von Gersdorff *et al*, 2000). While Smad7 is induced during androgen-withdrawal-induced apoptosis in prostate cells (Brodin *et al*, 1999), Smad7 induction has also been shown to antagonise directly TGF-*β*1 signalling (Whitman, 1997). Considering the evidence that Smad7-mediated apoptosis functionally involves inhibition of NF-*κ*B nuclear signalling (Lallemand *et al*, 2001; Schiffer *et al*, 2001) and in view of the present observations that I*κ*B*α* is upregulated within 6–24 h, preceding the induction of Smad7 (observed at 48 h of treatment), one may suggest that doxazosin may indeed have inhibitory effects on NF-*κ*B signalling pathway, by inducing I*κ*B*α*, an action that subsequently triggers apoptosis.

TIEG1 is an early TGF-*β*1 upregulated transcription factor that has been documented to regulate apoptosis in several tumour cell lines. The role of this nuclear factor has not yet been characterised in prostate growth, but it might potentially involve the activation of proapoptotic mechanisms, such as the downregulation of bcl-2 as reported in lung epithelial cells (Chalaux *et al*, 1999).

The *α*1-adrenoceptor antagonists doxazosin and terazosin share structural similarities with particular TKIs owing to the presence of a quinazoline ring (Figure 1). It is thus of major interest that several quinazoline-based chemicals have been demonstrated to compete with ATP binding to protein tyrosine kinases to inhibit various intracellular signalling pathways (Rewcastle *et al*, 1995). The mechanism of action for quinazoline-based TKI involves prevention of the transmembrane tyrosine kinase phosphorylation by competing with the ATP-binding site on the receptor (Arteaga *et al*, 1997). The presence of this component may provide additional valuable insights into the molecular mechanisms for *α*1-adrenoceptor antagonist-mediated apoptotic action in tumour cells (Anglin *et al*, 2002). Considering this evidence, one can speculate on the potential function of the intrinsic quinazoline component to confer partial TKI activity to these drugs, a unique structural characteristic with potentially significant pharmacological dimensions that warrant further investigation. Moreover, a potential antiangiogenic effect of terazosin and doxazosin against prostate tumours has been demonstrated in both *in vitro* and in clinical prostate specimens (Chon *et al*, 1999; Keledjian *et al*, 2001; Keledjian and Kyprianou, 2003). This action is believed to be mediated by quinazoline-induced anoikis and inhibition of cell invasion (Keledjian and Kyprianou, 2003).

In conclusion, we have demonstrated that doxazosin-mediated apoptosis in prostate cancer cells involves activation of latent apoptotic machinery via effectors of TGF-*β*1 signalling. In addition, this initial molecular dissection revealed that an inhibitory pathway involving NF-*κ*B is triggered by this quinazoline-based *α*1-adrenoceptor antagonist. Several signalling mechanisms are likely to be involved in a molecular crosstalk, such as the Smad activation and inhibition of the antiapoptotic effects of NF-*κ*B. Ongoing studies are focused on further characterisation of these pathways and the functional significance of the overexpressed genes in specimens from doxazosin-treated patients. This will provide the molecular basis for assessing the potential therapeutic significance of quinazoline monotherapy in androgen-independent prostate cancer.

## References

[bib1] Anglin I, Glassman D, Kyprianou N (2002) Induction of prostate apoptosis by *α*1-adrenoceptor antagonists: mechanistic significance of the quinazoline component. Prostate Cancer Prostatic Dis 5: 88–95 12496995 10.1038/sj.pcan.4500561

[bib2] Arteaga CL, Ramsey TT, Shawver LK, Guyer CA (1997) Unliganded epidermal growth factor receptor dimerization induced by direct interaction of quinazolines with the ATP binding site. J Biol Chem 272: 23247–23254 9287333 10.1074/jbc.272.37.23247

[bib3] Baldwin Jr AS (1996) The NF-*κ*B and I*κ*B proteins: new discoveries and insights. Annu Rev Immunol 14: 649–681 8717528 10.1146/annurev.immunol.14.1.649

[bib4] Benning CM, Kyprianou N (2002) Quinazoline-derived alpha1-adrenoceptor antagonists induce prostate cancer cell apoptosis via an alpha1-adrenoceptor-independent action. Cancer Res 62: 597–602 11809715

[bib5] Biswas DK, Dai SC, Cruz A, Weiser B, Graner E, Pardee AB (2001) The nuclear factor kappa B (NF-kappa B): a potential therapeutic target for estrogen receptor negative breast cancers. Proc Natl Acad Sci USA 98: 10386–10391 11517301 10.1073/pnas.151257998 PMC56970

[bib6] Boyle P (2000) Prostate cancer: evolution of an epidemic of unknown origin. In Prostate Cancer Denis L (ed) pp 5–11. Heidelberg: Springer-Verlag

[bib7] Brodin G, ten Dijke P, Funa K, Heldin CH, Landstrom M (1999) Increased smad expression and activation are associated with apoptosis in normal and malignant prostate after castration. Cancer Res 59: 2731–2738 10363999

[bib8] Bruckheimer EM, Kyprianou N (2000) Apoptosis in prostate carcinogenesis. A growth regulator and a therapeutic target. Cell Tissue Res 301: 153–162 10928288 10.1007/s004410000196

[bib9] Caine M (1988) Alpha-adrenergic mechanisms in dynamics of benign prostatic hypertrophy. Urology 32: 16–20 2462300

[bib10] Catalona WJ, Smith DS, Ratliff TL, Basler JW (1993) Detection of organ confined prostate cancer is associated through prostate-specific antigen-based screening. JAMA 270: 948–954 7688438

[bib11] Chalaux E, Lopez-Rovira T, Rosa JL, Pons G, Boxer LM, Bartrons R, Ventura F (1999) A zinc-finger transcription factor induced by TGF-beta promotes apoptotic cell death in epithelial Mv1Lu cells. FEBS Lett 457: 478–482 10471833 10.1016/s0014-5793(99)01051-0

[bib12] Chen W, Frank ME, Jin W, Wahl SM (2001) TGF-beta released by apoptotic T cells contributes to an immunosuppressive milieu. Immunity 14: 715–725 11420042 10.1016/s1074-7613(01)00147-9

[bib13] Chipuk JE, Bhat M, Hsing AY, Ma J, Danielpour D (2001) Bcl-xL blocks transforming growth factor-beta 1-induced apoptosis by inhibiting cytochrome *c* release and not by directly antagonizing Apaf-1-dependent caspase activation in prostate epithelial cells. J Biol Chem 276: 26614–26621 11320089 10.1074/jbc.M100913200

[bib14] Chon JK, Borkowski A, Partin AW, Isaacs JT, Jacobs SC, Kyprianou N (1999) Alpha 1-adrenoceptor antagonists terazosin and doxazosin induce prostate apoptosis without affecting cell proliferation in patients with benign prostatic hyperplasia. J Urol 161: 2002–2008 10332490

[bib15] Cohen G (1997) Caspases: the executioners of apoptosis. Biochem J 326: 1–16 9337844 10.1042/bj3260001 PMC1218630

[bib16] Crawford ED, Eisenberger MA, McLeod DG, Spaulding JT, Benson R, Dorr FA, Blumenstein BA, Davis MA, Goodman PJ (1989) A controlled trial of leuprolide with and without flutamide in prostatic carcinoma. N Engl J Med 321: 419–424 2503724 10.1056/NEJM198908173210702

[bib17] DeCaestecker MP, Piek E, Roberts AB (2000) Role of transforming growth factor-beta signaling in cancer. J Natl Cancer Inst 92: 1388–1402 10974075 10.1093/jnci/92.17.1388

[bib18] Gasparian AV, Yao YJ, Kowalcyk D, Lyakh LA, Karseladze A, Siaga TJ, Budunova RV (2002) The role of IKK in constitutive activation of NF-*κ*B transcription factor in prostate carcinoma cells. J Cell Sci 115: 141–151 11801732 10.1242/jcs.115.1.141

[bib19] Glassman DT, Chon JK, Borkowski A, Jacobs SC, Kyprianou N (2001) Combined effect of terazosin and finasteride on apoptosis, cell proliferation and transforming growth factor-*β* expression in benign prostatic hyperplasia. Prostate 46: 45–51 11170131 10.1002/1097-0045(200101)46:1<45::aid-pros1007>3.0.co;2-u

[bib20] Greenlee RT, Murray T, Bolden S, Wingo PA (2000) Cancer statistics. CA: Cancer J Clin 50: 7–33 10735013 10.3322/canjclin.50.1.7

[bib21] Hayashi H, Abdollah S, Qiu Y, Cai J, Xu YY, Grinnell BW, Richardson MA, Topper JN, Gimbrone Jr MA, Wrana JL, Falb D (1997) The MAD-related protein Smad7 associates with the TGF-beta receptor and functions as an antagonist of TGF-beta signaling. Cell 89: 1165–1173 9215638 10.1016/s0092-8674(00)80303-7

[bib22] Hefferan TE, Subramaniam M, Khosla S, Riggs BL, Spelsberg TC (2000) Cytokine-specific induction of the TGF-beta inducible early gene (TIEG): regulation by specific members of the TGF-beta family. J Cell Biochem 78: 380–390 10861837 10.1002/1097-4644(20000901)78:3<380::aid-jcb4>3.0.co;2-l

[bib23] Huang S, DeGuzman A, Bucana CD, Fidler IJ (2000) Nuclear factor-kappaB activity correlates with growth, angiogenesis, and metastasis of human melanoma cells in nude mice. Clin Cancer Res 6: 2573–2581 10873114

[bib24] Huang S, Pettaway CA, Uehara H, Bucana CD, Fidler IJ (2001) Blockade of NF-kappaB activity in human prostate cancer cells is associated with suppression of angiogenesis, invasion, and metastasis. Oncogene 20: 4188–4197 11464285 10.1038/sj.onc.1204535

[bib25] Isaacs JT (1994) Role of androgens in prostatic cancer. Vitam Horm 49: 433–502 7810075 10.1016/s0083-6729(08)61152-8

[bib26] Keledjian K, Borkowski A, Kim G, Isaacs JT, Jacobs SC, Kyprianou N (2001) Reduction of human prostate tumor vascularity by *α*1-adrenoreceptor antagonist terazosin. Prostate 48: 71–78 11433417 10.1002/pros.1083

[bib27] Keledjian K, Kyprianou N (2003) Anoikis-induction by quinazoline-based *α*1-adrenoceptor antagonists in prostate cancer cells: antagonistic effect of bcl-2. J Urol 169: 1150–1156 12576871 10.1097/01.ju.0000042453.12079.77

[bib28] Kim SK, Fan Y, Papadimitrakopoulou V, Clayman G, Hittelman WN, Hong WK, Lotan R, Mao L (1996) DPC4 a candidate tumor suppressor gene, is altered infrequently in head and neck squamous cell carcinoma. Cancer Res 56: 2519–2521 8653689

[bib29] Kirby R (1996) Doxazosin in the treatment of obstruction of the lower urinary tract. In Textbook of Benign Prostatic Hyperplasia Kirby R, McConnell J, Fitzpatrick J, Roehrborn C and Boyle P (eds) pp 287–293. Oxford: ISIS Medical Media

[bib31] Kyprianou N, Benning CM (2000) Suppression of human prostate cancer cell growth by alpha 1-adrenoreceptor antagonists doxazosin and terazosin via induction of apoptosis. Cancer Res 60: 4550–4555 10969806

[bib32] Kyprianou N, Isaacs JT (1989) Expression of transforming growth factor-beta in the rat ventral prostate during castration-induced programmed cell death. Mol Endocrinol 3: 1515–1522 2608047 10.1210/mend-3-10-1515

[bib33] Lallemand F, Mazars A, Prunier C, Bertrand F, Kornprost M, Gallea S, Roman-Roman S, Cherqui G, Atfi A (2001) Smad7 inhibits the survival nuclear factor kappaB and potentiates apoptosis in epithelial cells. Oncogene 20: 879–884 11314022 10.1038/sj.onc.1204167

[bib34] Liu F, Pouponnot C, Massague J (1997) Dual role of the Smad4/DPC4 tumor suppressor in TGFbeta-inducible transcriptional complexes. Genes Dev 11: 3157–3167 9389648 10.1101/gad.11.23.3157 PMC316747

[bib35] Massague J (2000) How cells read TGF-beta signals. Nat Rev Mol Cell Biol 1: 169–178 11252892 10.1038/35043051

[bib36] Massague J, Blain SW, Lo RS (2000) TGF-beta signaling in growth control, cancer, and heritable disorders. Cell 103: 295–309 11057902 10.1016/s0092-8674(00)00121-5

[bib37] Mazars A, Lallemand F, Prunier C, Marais J, Ferrand N, Pessah M, Cherqui G, Atfi A (2001) Evidence for a role of the JNK cascade in Smad7-mediated apoptosis. J Biol Chem 276: 36797–36803 11477069 10.1074/jbc.M101672200

[bib38] Pajonk F, Pajonk K, McBride WH (1999) Inhibition of NF-kappaB, clonogenicity, and radiosensitivity of human cancer cells. J Natl Cancer Inst 91: 1956–1960 10564680 10.1093/jnci/91.22.1956

[bib39] Palayoor ST, Youmell MY, Calderwood SK, Coleman CN, Price BD (1999) Constitutive activation of IkappaB kinase alpha and NF-kappaB in prostate cancer cells is inhibited by ibuprofen. Oncogene 18: 7389–7394 10602496 10.1038/sj.onc.1203160

[bib40] Raghavan D (1988) Non-hormone chemotherapy for prostate cancer: principles of treatment and application to the testing of new drugs. Semin Oncol 15: 371–389 3043671

[bib41] Rewcastle GW, Denny WA, Bridges AJ, Zhou H, Cody DR, McMichael A, Fry DW (1995) Tyrosine kinase inhibitors. 5. Synthesis and structure–activity relationships for 4-[(phenylmethyl)amino]- and 4-(phenylamino)quinazolines as potent adenosine 5′-triphosphate binding site inhibitors of the tyrosine kinase domain of the epidermal growth factor receptor. J Med Chem 38: 3482–3487 7658435 10.1021/jm00018a008

[bib42] Saha D, Datta PK, Beauchamp RD (2001) Oncogenic ras represses transforming growth factor-beta/Smad signaling by degrading tumor suppressor Smad4. J Biol Chem 276: 29531–29537 11371552 10.1074/jbc.M100069200

[bib43] Schiffer M, Bitzer M, Roberts IS, Kopp JB, ten Dijke P, Mundel P, Bottinger EP (2001) Apoptosis in podocytes induced by TGF-beta and Smad7. J Clin Invest 108: 807–816 11560950 10.1172/JCI12367 PMC200928

[bib44] Suh J, Faribourz P, Edeltein LC, Amenta PS, Zong W-X, Gelinas C, Rabson AB (2002) Mechanisms of constitutive NF-*κ*B activation in human prostate cancer cells. Prostate 52: 183–200 12111695 10.1002/pros.10082

[bib45] Tachibana I, Imoto M, Adjei PN, Gores GJ, Subramaniam M, Spelsberg TC, Urrutia R (1997) Overexpression of the TGF beta-regulated zinc finger encoding gene, TIEG, induces apoptosis in pancreatic epithelial cells. J Clin Invest 99: 2365–2374 9153278 10.1172/JCI119418 PMC508075

[bib46] Von Gersdorff G, Susztak K, Rezvani F, Bitzer M, Liang D, Bottinger EP (2000) Smad3 and Smad4 mediate transcriptional activation of the human Smad7 promoter by transforming growth factor beta. J Biol Chem 275: 11320–11326 10753944 10.1074/jbc.275.15.11320

[bib48] Whitman M (1997) Signal transduction. Feedback from inhibitory SMADs. Nature 389: 549–551 9335489 10.1038/39202

[bib49] Young RA, Brogden RM (1988) Doxazosin: a review of its pharmacodynamic and pharmokinetic properties, and therapeutic efficacy in mild or moderate hypertension. Drugs 33: 525–541 10.2165/00003495-198835050-00003 2899495

